# Using an Incidental Separated Segment of the Socket Wall as an Autogenous Block: A Lesson Learned Case Report of Three‐Dimensional Bone Reconstruction in the Anterior Maxilla

**DOI:** 10.1002/ccr3.72514

**Published:** 2026-04-10

**Authors:** Mehdi Ekhlasmandkermani, Mohammadreza Talebi Ardakani, Fatemeh Goudarzimoghaddam, Emir Ilkerli

**Affiliations:** ^1^ Periodontist, Member of the Editorial Board of the Oral Health and Oral Epidemiology Journal, School of Dentistry Kerman University of Medical Sciences Kerman Iran; ^2^ Department of Periodontics, School of Dentistry Shahid Beheshti University of Medical Sciences Tehran Iran; ^3^ Department of Periodontics, School of Dentistry Babol University of Medical Sciences Mazandaran Iran; ^4^ Department of Orthopedics and Traumatology Ostemed Klinik Bremervörde Bremervörde Germany

**Keywords:** alveolar bone grafting, autogenous bone graft, block bone graft, dental implant, tooth extraction, tooth socket

## Abstract

This case report discusses a patient who was referred for the right maxillary central incisor extraction, with subsequent plans for implant replacement and reconstruction of the lateral incisor site. During the extraction of the tooth, a fragment of the distal wall of the socket was fractured. Given that the fractured piece resembled an autogenous block and was amenable to being screwed, it was subsequently positioned to address the coronal defect in the lateral incisor area following the implant placement. Small‐sized cortical allograft powder was utilized to fill the area beneath the block, followed by coverage with a barrier membrane before closing the flap. After 4 months of reconstruction, clinical observations indicated successful bone reconstruction in both horizontal and vertical dimensions. Furthermore, the tooth socket, which had previously lost its distal portion, demonstrated promising and satisfactory reconstruction. The implant placed in the central incisor successfully osseointegrated without any complications. Due to the restricted space for the placement of two implants, the patient has been referred for further treatment plan involves a fixed prosthesis supported by implants in the central area, complemented by cantilevers in the lateral incisor site. The presented case demonstrates that when the bone dimensions of the incidentally separated fragments obtained during tooth extraction are adequate, and their suitability for screw fixation is confirmed, these fragments can effectively serve as autogenous blocks, even in vertical reconstruction.

## Key Clinical Message

Tooth extraction might result in the detachment of fragments from the socket walls. These fragments can serve as autogenous bone, offering potential benefits in surgical applications. In cases where the separated bone fragment is suitable for screwing, it can serve as an autogenous block for three‐dimensional bone reconstruction.

## 1 Introduction

Dimensional changes in bone following tooth extraction are a natural occurrence and can be attributed to both biological and mechanical factors [1]. Following Wolff's law, the structure and mass of bone are influenced by mechanical stress and strain. Consequently, in the absence of teeth and the associated forces, there is a potential for bone resorption to take place [2]. The remodeling of the alveolar bone ridge following tooth extraction results in noteworthy dimensional changes in both the horizontal and vertical planes [3, 4, 5, 6, 7, 8]. Research indicates that the resorption rate is more pronounced in the horizontal dimension compared to the vertical dimension [5, 8]. However, it is essential to recognize that reconstruction and management of complications associated with vertical bone defects present greater challenges post‐surgery [9, 10].

Over the past decades, various techniques have been developed for the vertical reconstruction of each defect, tailored to the specific type and severity of the defect. Notable methods in this field include distraction osteogenesis, block grafting (onlays or inlays, inter‐positional grafts), guided bone regeneration (GBR) utilizing non‐absorbable membranes, the application of titanium mesh, and the implementation of tent screws [10, 11, 12, 13, 14, 15, 16, 17, 18, 19]. While there remains a lack of consensus regarding the most suitable method for vertical reconstruction, it is crucial to consider two fundamental factors when implementing any vertical reconstruction technique. Firstly, the presence of supportive bony walls (bone peaks) and the second is the availability of appropriate soft tissue for achieving a tension‐free closure [20, 21, 22].

The characteristics of the bone peaks within the edentulous area play a crucial role in determining the regeneration potential of the defect, particularly in relation to the chosen reconstruction technique. In cases with wide vertical defects, where a considerable distance exists between the bone peaks, inadequate angiogenesis often hinders the regenerative potential [20, 21, 23]. In circumstances where the potential for bone regeneration is diminished, utilizing autogenous bone, whether as particles or blocks, may be the preferred option to enhance osteogenic properties and promote osteoinductivity [24]. In the context of vertical reconstructions, achieving primary closure presents greater challenges than horizontal reconstructions. Utilizing autogenous blocks may offer a reduced risk of early wound exposure compared to non‐absorbable membranes or titanium meshes [13, 25, 26, 27]. Furthermore, autogenous blocks demonstrate superior space preservation and diminished late resorption relative to autogenous bone particles, highlighting their advantages in vertical reconstruction procedures [28].

In certain cases, a segment of alveolar bone may be removed along with the tooth root during tooth extraction. When this fragment can be securely repositioned at the defect site, it can serve as an autogenous block, providing a gold‐standard option for horizontal and vertical bone reconstruction.

This study presents a simple and minimally invasive approach known as the socket wall autogenous bone block approach for vertical reconstruction in conjunction with tooth extraction. This method has the potential to yield promising results in clinical practice.

## 2 Case History

**FIGURE 1:**
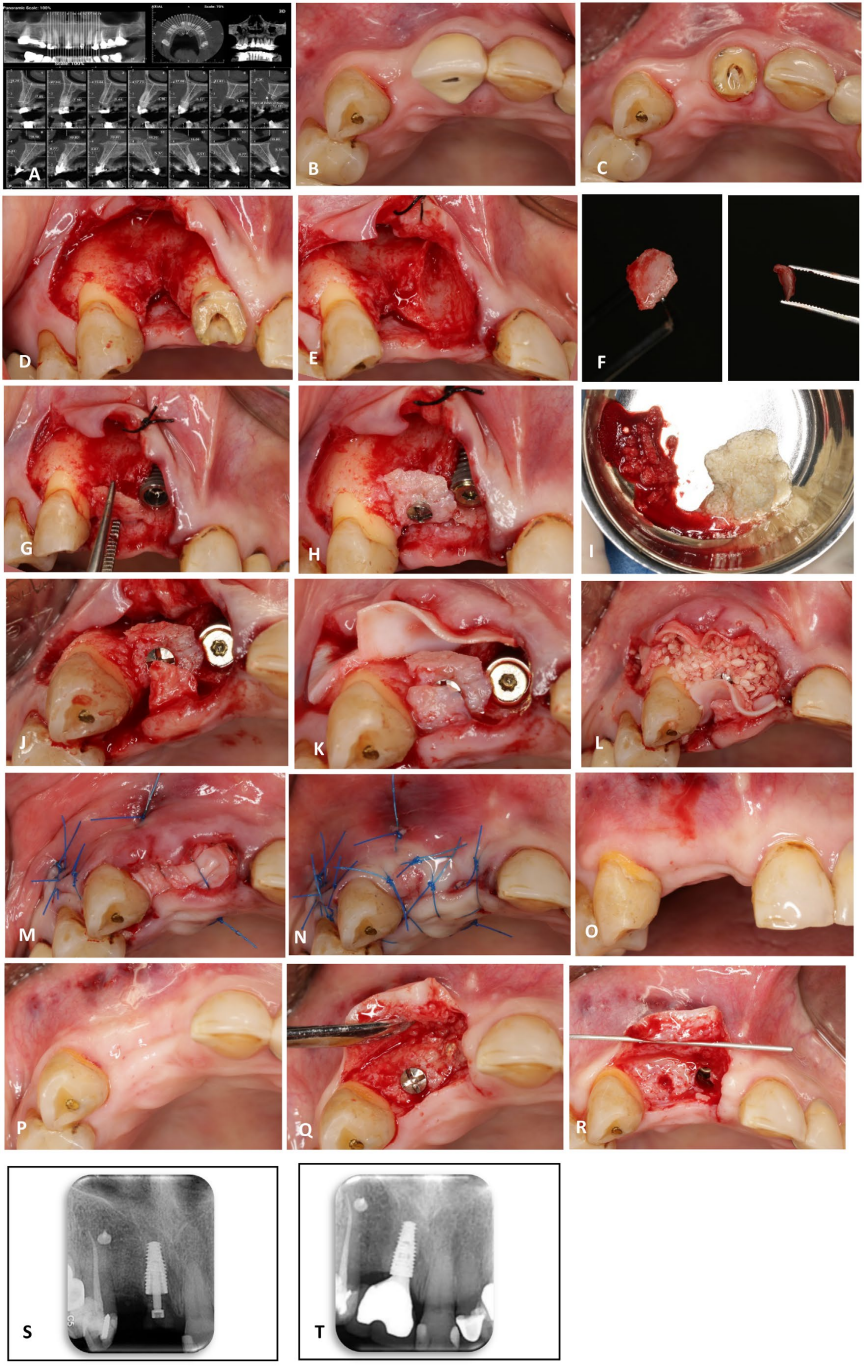
(A) The CBCT reveals a noticeable vertical resorption in the lateral incisor position. (B) Occlusal view featuring a central incisor crown. (C) The occlusal view following the central incisor crown removal. Unfortunately, retreatment of the root canal was not feasible, and the patient has been referred for implant replacement of the central tooth. (D) The vertical resorption view. The flap with a vertical release was meticulously prepared at the distal aspect of the canine. Notably, the interproximal papilla between the central incisors was preserved intact. (E) Central incisor extraction. In addition to the tooth extraction, a portion of the distal socket wall was also removed, resulting in a horizontal defect being added to the existing vertical defect. (F) Socket wall bone block. A corticocancellous bone fragment was removed from the central incisor root. (G) The implant was placed in the position of the central incisor, and the bone block was assessed for placement at the vertical resorption site. (H) Bone block fixation. The bone block was fixed vertically using a screw, resting on the remaining bone peaks in both the mesial and distal directions. (I)Bone material preparation. A mixture of cortical bone powder and autogenous bone particles was prepared with the patient's blood. (J) Given the sufficient thickness of soft tissue in the palatal area, a vascularized connective tissue graft (CTG) pedicle was prepared to match the size of the bone block cover. (K) Membrane fixation. The first membrane was secured in the apical area using a bone tac. (L) Bone material filling. The second membrane was positioned in the apical area, and the bone material mixture was used to fill the space beneath the bone block. (M) First layer suturing. The first layer of sutures was applied using an internal horizontal mattress technique. At this stage, an effort was made to position the pedicle CTG correctly on the membrane. Simultaneously, the vertical release was closed. (N) Second layer suturing. Simple interrupted sutures were used to bring the flap edges together. No attempt was made to completely close the socket orifice at the central incisor site to utilize the potential of the socket for spontaneous closure of the orifice. (O) Buccal view after 4 months. The healing process progressed uneventfully and the midline papilla was maintained in a proper position. (P) Occlusal view after 4 months of healing. The socket orifice was completely sealed in the central incisor site. (Q) Flap elevation. The new bone encompassed both the vertical defect and the implant periphery in a three‐dimensional manner. (R) Screw removal. The bone screw was removed, and the cover screw was replaced with a healing abutment. (S, T) The preapical view demonstrates the clinical outcomes following the gingival former placement and 8 months after prosthetic rehabilitation.

A 67‐year‐old non‐smoker female patient with stable systemic health was referred for the extraction of a central incisor that was deemed non‐restorable due to endodontic complications. Cone beam computed tomography (CBCT) revealed vertical bone loss in the adjacent lateral tooth incisor (Figure 1A–C). During the extraction procedure, an unintended fragmentation of a bony segment occurred. The fragment segment thickness varies across different areas, with an average measurement of 1.5 mm. Additionally, the width is approximately 5 mm, while the length is around 7 mm. All sharp edges have been carefully trimmed to minimize the risk of soft tissue injury (Figure 1E,F).

The characteristic of the separated bone fragment is that it consists of both cortical and cancellous tissue, allowing for secure fixation with screws. Once adequately secured, no movement must be detected in the fixed fragment, with the adjacent bony peaks serving as stabilizing stops (Figure 1G,H). The procedures were conducted in a private practice by a single practitioner. The participant in the study provided informed consent and was fully aware of the treatment protocol before being admitted to the private clinic.

## 3 Investigation and Treatment

Following a thorough explanation of the reconstruction technique and obtaining the patient's consent, a recommendation was made to administer amoxicillin 500 mg the day before surgery, in accordance with standard treatment protocols [29]. On surgery day, a 0.2% chlorhexidine mouthwash (Iran Najo Chlorhexidine 0.2% Mouthwash, 250 mL; Iran) was used for 1 min. During the tooth extraction procedure, it was noted that due to the ankylosis of the central incisor root, a significant portion of the socket wall adjacent to the lateral incisor was excised along with the root (Figure 1D,E). Given the substantial size of the removed cortical bone (The fragment segment thickness varies across different areas, with an average measurement of 1.5 mm. Additionally, the width is approximately 5 mm, while the length is around 7 mm.), it was carefully separated from the root through precise movements of the surgical blade (Figure 1F). All sharp edges have been carefully trimmed to minimize the risk of soft tissue injury.

The presence of cortical bone enhanced the secure fixation of the component (Figure 1G). Consequently, the separated fragment was utilized as an autogenous block. To improve accessibility, a vertical release was conducted at the distal of the canine tooth.

A quantity of autogenous particles was collected from the drilling site within the socket and from an adjacent area and placed into a galipot to create a bone‐conditioned medium [30]. Following 15 min, the rehydrated pure cortical powder of Allograft was incorporated into the galipot containing the autogenous particles. This final mixture was subsequently prepared for GBR (Figure 1I).

Following the placement of the implant in the central incisor position, the autogenous block was immediately secured using a single screw positioned at the coronal aspect of the adjacent vertical defect. This fixation method effectively connected the bone peaks adjacent to the defect while also allowing for the maintenance of space required for vertical reconstruction by extending horizontally in the lateral incisor position (Figure 1H). The area beneath the autogenous block was appropriately filled with a combination of small particles of pure cortical allograft material (FDBA, 500–1000 μm; Iranian Tissue Product Company) and autogen particles. A xenograft membrane (Tutogen Medical GmbH; Germany) was utilized to completely cover the defect as well as the bone particles in both the buccal and palatal directions (Figure 1K). Additionally, to minimize the risk of wound exposure and enhance the soft tissue height at the defect site, a connective tissue graft (CTG) pedicle from the palatal flap was employed (Figure 1J). An internal horizontal mattress suture (Nylon 5–0, Nasj Tebb Keyhan, Iran) alleviated tension along the flap edge (Figure 1M). The closure of the flap was subsequently achieved using simple sutures (Figure 1N). It was recommended that antibiotic therapy continue for 1 week following the surgical procedure. The removal of sutures 2 weeks post‐surgery indicated that healing occurred without complications during this period.

## 4 Result and Conclusion

Four months following the initial surgical procedure, the patient was referred for implant uncover (Figure 1O–S). Eight months clinical and radiographic evaluation after prosthetic delivery indicated successful vertical and horizontal reconstruction in the lateral incisor and the coronal regions of the implant located at the central incisor site (Figure 1T).

The Socket Wall Autogenous Bone Block Technique represents a straightforward and minimally invasive vertical and horizontal bone reconstruction method. This report demonstrates that bone fragments retrieved during tooth extraction can effectively serve as autogenous blocks for bone regeneration under suitable conditions. Using a separated alveolar bone fragment as an autogenous block presents several advantages. Notably, this technique eliminates the need for a donor site, enhancing the patient experience. Additionally, it promotes improved angiogenesis potential by ensuring maximum contact with the remaining bone peaks. This approach also facilitates optimal space maintenance or creation and minimizes the risk of wound exposure, which enhances the possibility of successful reconstruction outcomes. Providing the three essential properties of autogenous bone—osteogenic, osteoinductive, and osteoconductive—enhances the predictability and efficiency of bone reconstruction, making it a low‐risk approach for vertical and horizontal defect management. However, tooth extraction procedures should be conducted with a conservative approach, prioritizing minimal trauma to the surrounding bone walls to prevent any fractures. It is generally advised against the removal of fragments from the socket wall, specifically for the purpose of reconstructing adjacent defects.

## 5 Discussion

Vertical bone regeneration presents significant challenges in intra‐oral reconstruction. Selecting the most suitable technique largely depends on the defect morphology and exhibits considerable variability. In recent years, innovative methods such as the shell technique, utilizing autogenous cortical plates or allografts, have demonstrated impressive outcomes during the healing process. Nevertheless, the benefits of employing autogenous blocks remain well‐recognized as the gold standard in bone regeneration treatment [31].

Autogenous bone blocks can be obtained both intraorally and from extraoral sources. Common intraoral donor sites include the tuberosity, symphysis, and ramus [32, 33]. Although intraoral sources offer high‐quality bone, several limitations, such as finite availability of resources, patient discomfort, and extended surgical procedure times, may affect the selection of this approach for vertical reconstructions [23, 34, 35]. To address this issue, recent literature has explored the application of bone at the same defect site as a less invasive approach. One such article, titled “In‐Situ Shell Technique,” demonstrates that if it is feasible to separate segments of the bone defect walls, these sections can be repositioned to serve as new walls, thereby creating additional space by altering block positions [36]. These blocks serve a dual purpose by facilitating space creation while also demonstrating osteogenic and osteoinductive properties. Additionally, they play a crucial role in preventing soft tissue contraction around the bone particles [20, 37]. Space creation or maintenance is a fundamental aspect of the PASS principles, which were introduced in 2006 to enhance the predictability of reconstruction outcomes [20].

Based on the in situ technique, utilizing a bone fragment separated during tooth extraction can serve as a tent for reconstructing the existing defect. In vertical bone reconstructions, the probability of soft tissue collapse is higher than in horizontal reconstructions, and the predictability of vertical reconstruction is more difficult. The broken fragment acted as a tent to reduce the probability of vertical soft tissue collapse [23, 38].

In this article, the separated bone fragment contained a cortical component and was suitable for fixation. It was securely positioned in the coronal aspect of the vertical defect to leverage its osteogenic, osteoinductive, and osteoconductive properties effectively. A bony fragment that has become inadvertently detached due to its thickness exceeding 1 mm may experience limited and permanent resorption throughout the period required for the intended defect repair. The outcomes assessed 4 months post‐surgery indicate a complete reconstruction of the defect in both vertical and horizontal dimensions.

The extent to which autogenous blocks influence new bone formation has consistently been a key inquiry in the field of reconstruction utilizing these blocks. This issue was addressed in an article by Urban et al. in 2022 that introduced different vertical reconstruction techniques [23]. A review of a study conducted by Gray in 1979 indicates that the osteogenic properties of autologous bone blocks are significantly influenced by the quantity of viable cells within the block structure and their survival rates after 3 to 5 days following implantation at the defect site [39]. Plasma diffusion can only sustain the viability of cells within the bone block for a restricted time. Therefore, the prolonged survival of these cells and, subsequently, the osteogenic potential of the autologous block are fundamentally reliant on the process of new angiogenesis and the infiltration of blood capillaries into the block. In conclusion, it can be observed that a stronger connection between the autologous block and the source of vascular supply (the remaining bone walls at the defect site) significantly enhances the potential for angiogenesis. This process is fundamental to achieving successful outcomes when using autologous blocks.

This article concludes that the autogenous block significantly contributed to the reconstruction of the defect as an osteogenic block, as maximum contact was established between the block and the remaining bone peaks in the coronal aspect of the defect.

Furthermore, it is essential to highlight that pure cortical allograft particles exclusively occupied the area generated beneath the autogenous block. The application of cortical allograft powders has been previously established in situations where greater space maintenance is required [20].

This suggests that autogenous chips are not necessary for defect reconstruction in the presence of an autogenous block and even without comprehensive coverage of a defect. Allograft particles can yield favorable outcomes within a short period.

## Author Contributions

**Mehdi Ekhlasmandkermani:** conceptualization, investigation, methodology, project administration, supervision, visualization, writing – original draft, writing – review and editing. **Mohammadreza Talebi Ardakani:** supervision, writing – review and editing. **Fatemeh Goudarzimoghaddam:** investigation, project administration, supervision, visualization, writing – original draft, writing – review and editing. **Emir Ilkerli:** writing – review and editing.

## Funding

The authors have nothing to report.

## Ethics Statement

The authors have nothing to report.

## Consent

Written informed consent was obtained from the patient for the publication of this study and its associated images.

## Conflicts of Interest

The authors declare no conflicts of interest.

## Data Availability

The data that support the findings of this study are available from the corresponding author upon reasonable request.

## References

[1] S. E. Udeabor , A. Heselich , S. Al‐Maawi , A. F. Alqahtani , R. Sader , and S. Ghanaati , “Current Knowledge on the Healing of the Extraction Socket: A Narrative Review,” Bioengineering (Basel) 10, no. 10 (2023): 1145. 37892875 10.3390/bioengineering10101145 PMC10604628

[2] J. Wolff , “Das Gesetz Der Transformation Der Knochen,” Deutsche Medizinische Wochenschrift 19, no. 47 (1893): 1222–1224.

[3] G. E. Carlsson , “Changes in the Jaws and Facial Profile After Extractions and Prosthetic Treatment,” Transactions of the Royal Schools of Dentistry Stockholm and Umea 12 (1967): 1–29. 5231217

[4] G. E. Carlsson , N. Ragnarson , and P. Astrand , “Changes in Height of the Alveolar Process in Edentulous Segments. A Longitudinal Clinical and Radiographic Study of Full Upper Denture Cases With Residual Lower Anteriors,” Odontologisk Tidskrift 75, no. 3 (1967): 193–208. 5231586

[5] L. Schropp , A. Wenzel , L. Kostopoulos , and T. Karring , “Bone Healing and Soft Tissue Contour Changes Following Single‐Tooth Extraction: A Clinical and Radiographic 12‐Month Prospective Study,” International Journal of Periodontics & Restorative Dentistry 23, no. 4 (2003): 313–323. 12956475

[6] J. Pietrokovski and M. Massler , “Residual Ridge Remodeling After Tooth Extraction in Monkeys,” Journal of Prosthetic Dentistry 26, no. 2 (1971): 119–129. 4996978 10.1016/0022-3913(71)90041-2

[7] J. Pietrokovski and M. Massler , “Ridge Remodeling After Tooth Extraction in Rats,” Journal of Dental Research 46, no. 1 (1967): 222–231. 5226389 10.1177/00220345670460011501

[8] F. Van der Weijden , F. Dell'Acqua , and D. E. Slot , “Alveolar Bone Dimensional Changes of Post‐Extraction Sockets in Humans: A Systematic Review,” Journal of Clinical Periodontology 36, no. 12 (2009): 1048–1058. 19929956 10.1111/j.1600-051X.2009.01482.x

[9] S. Bernstein , J. Cooke , P. Fotek , and H. L. Wang , “Vertical Bone Augmentation: Where Are We Now?,” Implant Dentistry 15, no. 3 (2006): 219–228. 16966894 10.1097/01.id.0000226824.39526.71

[10] I. A. Urban , E. Montero , A. Monje , and I. Sanz‐Sánchez , “Effectiveness of Vertical Ridge Augmentation Interventions: A Systematic Review and Meta‐Analysis,” Journal of Clinical Periodontology 46, no. 21 (2019): 319–339. 30667522 10.1111/jcpe.13061

[11] S. J. Froum , E. S. Rosenberg , N. Elian , D. Tarnow , and S. C. Cho , “Distraction Osteogenesis for Ridge Augmentation: Prevention and Treatment of Complications. Thirty Case Reports,” International Journal of Periodontics & Restorative Dentistry 28, no. 4 (2008): 337–345. 18717372

[12] M. Chiapasco , R. Brusati , and P. Ronchi , “Le Fort I Osteotomy With Interpositional Bone Grafts and Delayed Oral Implants for the Rehabilitation of Extremely Atrophied Maxillae: A 1‐9‐Year Clinical Follow‐Up Study on Humans,” Clinical Oral Implants Research 18, no. 1 (2007): 74–85. 17224027 10.1111/j.1600-0501.2006.01287.x

[13] M. Chiapasco , M. Zaniboni , and L. Rimondini , “Autogenous Onlay Bone Grafts vs. Alveolar Distraction Osteogenesis for the Correction of Vertically Deficient Edentulous Ridges: A 2‐4‐Year Prospective Study on Humans,” Clinical Oral Implants Research 18, no. 4 (2007): 432–440. 17501979 10.1111/j.1600-0501.2007.01351.x

[14] C. H. Hämmerle and R. E. Jung , “Bone Augmentation by Means of Barrier Membranes,” Periodontology 2000 33 (2003): 36–53. 12950840 10.1046/j.0906-6713.2003.03304.x

[15] I. Beitlitum , Z. Artzi , and C. E. Nemcovsky , “Clinical Evaluation of Particulate Allogeneic With and Without Autogenous Bone Grafts and Resorbable Collagen Membranes for Bone Augmentation of Atrophic Alveolar Ridges,” Clinical Oral Implants Research 21, no. 11 (2010): 1242–1250. 20572833 10.1111/j.1600-0501.2010.01936.x

[16] D. J. Leong , T. J. Oh , E. Benavides , K. Al‐Hezaimi , C. E. Misch , and H. L. Wang , “Comparison Between Sandwich Bone Augmentation and Allogenic Block Graft for Vertical Ridge Augmentation in the Posterior Mandible,” Implant Dentistry 24, no. 1 (2015): 4–12. 25365652 10.1097/ID.0000000000000180

[17] P. Proussaefs and J. Lozada , “The Use of Intraorally Harvested Autogenous Block Grafts for Vertical Alveolar Ridge Augmentation: A Human Study,” International Journal of Periodontics & Restorative Dentistry 25, no. 4 (2005): 351–363. 16089043

[18] C. Tinti , S. Parma‐Benfenati , and G. Polizzi , “Vertical Ridge Augmentation: What Is the Limit?,” International Journal of Periodontics & Restorative Dentistry 16, no. 3 (1996): 220–229. 9084308

[19] F. Goudarzimoghaddam , M. Ekhlasmandkermani , B. Houshmand , and H. Sabri , “Internal Allo‐Cortical Tenting: A Modified Ridge Split Technique in Three‐Dimensional Ridge Augmentation,” Journal of Oral Implantology 50, no. 4 (2024): 384–390. 38895832 10.1563/aaid-joi-D-24-00004

[20] H. L. Wang and L. Boyapati , “PASS Principles for Predictable Bone Regeneration,” Implant Dentistry 15, no. 1 (2006): 8–17. 16569956 10.1097/01.id.0000204762.39826.0f

[21] U. M. Wikesjö , C. J. Kean , and G. J. Zimmerman , “Periodontal Repair in Dogs: Supraalveolar Defect Models for Evaluation of Safety and Efficacy of Periodontal Reconstructive Therapy,” Journal of Periodontology 65, no. 12 (1994): 1151–1157. 7877088 10.1902/jop.1994.65.12.1151

[22] I. A. Urban , A. Monje , J. Lozada , and H. L. Wang , “Principles for Vertical Ridge Augmentation in the Atrophic Posterior Mandible: A Technical Review,” International Journal of Periodontics & Restorative Dentistry 37, no. 5 (2017): 639–645. 28817126 10.11607/prd.3200

[23] I. A. Urban , E. Montero , E. Amerio , D. Palombo , and A. Monje , “Techniques on Vertical Ridge Augmentation: Indications and Effectiveness,” Periodontology 2000 93, no. 1 (2023): 153–182. 36721380 10.1111/prd.12471

[24] R. J. Miron , “Optimized Bone Grafting,” Periodontology 2000 94, no. 1 (2024): 143–160. 37610202 10.1111/prd.12517

[25] I. Milinkovic and L. Cordaro , “Are There Specific Indications for the Different Alveolar Bone Augmentation Procedures for Implant Placement? A Systematic Review,” International Journal of Oral and Maxillofacial Surgery 43, no. 5 (2014): 606–625. 24451333 10.1016/j.ijom.2013.12.004

[26] M. Roccuzzo , G. Ramieri , M. Bunino , and S. Berrone , “Autogenous Bone Graft Alone or Associated With Titanium Mesh for Vertical Alveolar Ridge Augmentation: A Controlled Clinical Trial,” Clinical Oral Implants Research 18, no. 3 (2007): 286–294. 17298495 10.1111/j.1600-0501.2006.01301.x

[27] I. A. Urban , M. H. A. Saleh , A. Ravidà , A. Forster , H. L. Wang , and Z. Barath , “Vertical Bone Augmentation Utilizing a Titanium‐Reinforced PTFE Mesh: A Multi‐Variate Analysis of Influencing Factors,” Clinical Oral Implants Research 32, no. 7 (2021): 828–839. 33786888 10.1111/clr.13755

[28] I. Rocchietta , M. Simion , M. Hoffmann , D. Trisciuoglio , M. Benigni , and C. Dahlin , “Vertical Bone Augmentation With an Autogenous Block or Particles in Combination With Guided Bone Regeneration: A Clinical and Histological Preliminary Study in Humans,” Clinical Implant Dentistry and Related Research 18, no. 1 (2016): 19–29. 25622713 10.1111/cid.12267

[29] A. O. Salgado‐Peralvo , M. V. Mateos‐Moreno , E. Velasco‐Ortega , J. F. Peña‐Cardelles , and N. Kewalramani , “Preventive Antibiotic Therapy in Bone Augmentation Procedures in Oral Implantology: A Systematic Review,” Journal of Stomatology Oral and Maxillofacial Surgery 123, no. 1 (2022): 74–80. 33493687 10.1016/j.jormas.2021.01.011

[30] M. B. Asparuhova , J. Caballé‐Serrano , D. Buser , and V. Chappuis , “Bone‐Conditioned Medium Contributes to Initiation and Progression of Osteogenesis by Exhibiting Synergistic TGF‐β1/BMP‐2 Activity,” International Journal of Oral Science 10, no. 2 (2018): 20. 29895828 10.1038/s41368-018-0021-2 PMC5997631

[31] A. Sakkas , F. Wilde , M. Heufelder , K. Winter , and A. Schramm , “Autogenous Bone Grafts in Oral Implantology‐Is It Still a Gold Standard? A Consecutive Review of 279 Patients With 456 Clinical Procedures,” International Journal of Implant Dentistry 3, no. 1 (2017): 23. 28573552 10.1186/s40729-017-0084-4 PMC5453915

[32] E. E. Keller , N. B. Van Roekel , R. P. Desjardins , and D. E. Tolman , “Prosthetic‐Surgical Reconstruction of the Severely Resorbed Maxilla With Iliac Bone Grafting and Tissue‐Integrated Prostheses,” International Journal of Oral & Maxillofacial Implants 2, no. 3 (1987): 155–165. 3325418

[33] C. M. Misch , “Comparison of Intraoral Donor Sites for Onlay Grafting Prior to Implant Placement,” International Journal of Oral & Maxillofacial Implants 12, no. 6 (1997): 767–776. 9425757

[34] S. Chavda and L. Levin , “Human Studies of Vertical and Horizontal Alveolar Ridge Augmentation Comparing Different Types of Bone Graft Materials: A Systematic Review,” Journal of Oral Implantology 44, no. 1 (2018): 74–84. 29135351 10.1563/aaid-joi-D-17-00053

[35] P. V. Giannoudis , H. Dinopoulos , and E. Tsiridis , “Bone Substitutes: An Update,” Injury 36, no. 3 (2005): S20–S27. 16188545 10.1016/j.injury.2005.07.029

[36] B. Houshmand , M. Talebi Ardakani , O. Amirinasab , et al., “In Situ Shell Technique for Edentulous Ridge Augmentation,” Clinical Advances in Periodontics 14, no. 1 (2024): 5–8. 36700457 10.1002/cap.10235

[37] B. Le , J. Burstein , and P. P. Sedghizadeh , “Cortical Tenting Grafting Technique in the Severely Atrophic Alveolar Ridge for Implant Site Preparation,” Implant Dentistry 17, no. 1 (2008): 40–50. 18332757 10.1097/ID.0b013e318166d503

[38] I. Urban , I. Sanz‐Sánchez , A. Monje , and E. Montero , “Complications and Treatment Errors in Peri‐Implant Hard Tissue Management,” Periodontology 2000 92, no. 1 (2023): 278–298. 37016554 10.1111/prd.12472

[39] J. C. Gray and M. W. Elves , “Early Osteogenesis in Compact Bone Isografts: A Quantitative Study of Contributions of the Different Graft Cells,” Calcified Tissue International 29, no. 3 (1979): 225–237. 117887 10.1007/BF02408085

